# Moving toward Smart Cities: Evaluation of the Self-Cleaning Properties of Si-Based Consolidants Containing Nanocrystalline TiO_2_ Activated by Either UV-A or UV-B Radiation

**DOI:** 10.3390/polym12112577

**Published:** 2020-11-02

**Authors:** José Santiago Pozo-Antonio, Daniel Noya-Pintos, Patricia Sanmartín

**Affiliations:** 1 Dpto. Enxeñaría dos Recursos Naturais e Medio Ambiente, Escola de Enxeñaría de Minas e Enerxía, Campus As Lagoas-Marcosende, University of Vigo, 36310 Vigo, Spain; 2 CINTECX, University of Vigo, 36310 Vigo, Spain; 3 Escola Superior de Conservación e Restauración de Bens Culturais de Galicia, 36002 Pontevedra, Spain; daniel.noya.pinto@gmail.com; 4 Departamento de Edafoloxía e Química Agrícola, Facultade de Farmacia, Universidade de Santiago de Compostela, 15782 Santiago de Compostela, Spain; patricia.sanmartin@usc.es

**Keywords:** self-cleaning, TiO_2_, consolidant, stone preservation, photocatalysis, smart city, natural and accelerated procedures

## Abstract

This study evaluated the self-cleaning ability and durability of Si-based consolidants (an ethyl silicate consolidant and a consolidant based on nanosized silica) spiked with nanocrystalline TiO_2_ activated by either UV-A radiation (spectral region between 340 and 400 nm, and main peak at 365 nm) or UV-B radiation (spectral region between 270 and 420 nm, and main peak at 310 nm). Granite samples were coated with consolidant, to which nanocrystalline TiO_2_ was added at different concentrations (0.5, 1, and 3%, by wt.). Diesel soot was then applied to the coated surfaces, and the samples were exposed to UV-A or UV-B radiation for 1650 h. The surface color changes, relative to the color of untreated granite, were determined every 330 h by color spectrophotometry. Slight color changes indicated a recovery of the reference color due to the degradation of the soot. The final surfaces of both the untreated and treated surfaces were compared by stereomicroscopy and scanning electron microscopy. The main findings were that: (1) In general, the consolidant containing nanosized silica induced the most intense photocatalytic activity. In the more compact xerogel coating formed by the nanosized silica, more TiO_2_ nanoparticles were available to interact with the radiation. (2) For all consolidant mixtures, soot degradation remained constant or decreased over time, except with ethyl silicate with 0.5 wt % TiO_2_ (no self-cleaning capacity). (3) Soot degradation increased with the concentration of TiO_2_. (4) The UV-B radiation was the most effective in terms of soot degradation, except for the surface coated with the ethyl silicate and 3% wt. TiO_2_.

## 1. Introduction

Urban designers are currently facing an important challenge involving the application of new technological and management strategies within the smart city model [1]. This model aspires to use technological solutions to improve the management and efficiency of the urban environment, with the ultimate aim of increasing urban sustainability [1]. Within this framework, preventing the deposition of fuel-derived soot on stone surfaces in both historic and contemporary buildings (e.g., by decomposition of the organic matter in the soot by applying UV-active nanocrystalline TiO_2_-based coatings) has been a challenge in the last decade [2,3,4,5,6]. The deposition of carbonaceous particles on surfaces in the urban-built fabric contributes to the formation of black crusts, which affect the durability and aesthetic appearance of the buildings [7,8]. On exposure to UV radiation (UV-A preferred to daylight [9]), TiO_2_ nanoparticles in the coatings become photocatalytic, thus enhancing the photo-decomposition of organic matter on the surfaces by redox reactions while also repelling water due to a hydrophobic effect [2,3,6]. This approach avoids the drawbacks associated with many cleaning procedures, such as chemical contamination, extraction of grains, and fusion of minerals [10]. 

Previous research findings on construction materials (e.g., stone, mortars, and bricks) coated with artificially synthesized substances, such as Printex-U [11], orange dye [12], red dyes [13], blue dye [4,12], and tobacco [14], rather than with real diesel exhaust particles, were taken into consideration in determining the following key factors regarding the cleaning efficacy of nanocrystalline TiO_2_ coatings:

**(1) The mineralogical composition of the nanoparticle TiO_2_ used.** TiO_2_ can occur in different crystalline forms: anatase, brookite, and rutile, with anatase exhibiting the highest photocatalytic activity [15,16]. Anatase is an indirect band gap semiconductor, while rutile and brookite are direct band gap semiconductors. Indirect band gap anatase exhibits a longer lifetime of photoexcited electrons and holes than direct band gap rutile and brookite [16]. Consequently, anatase has a lower average effective mass of photogenerated electrons and holes than rutile and brookite. The low effective mass suggests fast migration of photogenerated electrons and holes from the interior to surface of anatase particle. Therefore, the recombination rate of photogenerated charge carriers is lowest in anatase.

In a study with anatase alone and a commercial (78:14:8) anatase:rutile:amorphous-based dispersion, either in water or in ethylene glycol coated on Noto calcarenite and Carrara marble, better results (in terms of the degradation of rhodamine B) were observed on the surfaces coated only with anatase, due to the residual benzyl alcohol molecules anchored on the anatase nanoparticles [4].

**(b) The binder and the procedure used to disperse the TiO_2_.** Regarding the matrix in which the TiO_2_ nanoparticles are dispersed, coatings with TiO_2_ synthesized in water via polyol synthesis were sprayed onto samples of travertine and limestone, yielding satisfactory self-cleaning of rhodamine B, regardless of the number of applications [2]. In a study with nanocrystalline TiO_2_ dispersed in an aqueous suspension of an acrylic polymer on marble and limestone, a consolidant effect was observed in addition to photocatalytic (against methylene blue stains), hydrophobic, and biocidal effects [3]. In another study, nanocrystalline TiO_2_ was mixed with a commercial hydrophobic fluorpolymer on a limestone and exposed for a period of one year in the city of Bari (Italy) [5]. The components had a synergetic effect on water repellency and self-cleaning properties; however, the latter decreased gradually due to a reduction in photocatalysis from the coating surface, attributed to aging-related modification of the polymers [5]. Finally, satisfactory results were observed in a study of the degradation of rhodamine B on a travertine with an anatase coating prepared using a sol–gel method [6]. 

**(c) The type of radiation (daylight or UV radiation) applied to the coating in order to activate photocatalysis of the nanocrystalline TiO_2_**. Regarding UV radiation, as stated above, previous studies have reported the satisfactory response of UV-A radiation (in the spectral region 315–400 nm) in activating photocatalysis [2,3,6]. Natural and artificial solar light (including UV-A and UV-B radiation) also displayed efficient photocatalytic activity [4,5]. UV-B (spectral region: 280–315 nm) and UV-C (spectral region: 100–280 nm)-activated TiO_2_ have been used to a much lesser extent [17], and, as far as we are aware, never on coated stone surfaces in the urban fabric. The sun emits all three types of UV radiation, but the UV radiation that reaches the Earth’s surface comprises around 95% UV-A and 5% UV-B. Scientific research on the effect of UV-B radiation on the photocatalysis induced by TiO_2_ nanoparticles is justified in the context of global change, in which ozone depletion will lead to an increase in UV-B that reaches the Earth’s surface [18].

The application of nanocrystalline TiO_2_: anatase-based coatings has been proven successful in terms of the degradation of organic dyes. Thus, the addition of TiO_2_ nanoparticles to consolidant products can result in multifunctional coatings since, in addition to restoring the cohesion to disaggregated stone surfaces, these coatings also show self-cleaning property. As recommended by conservation professionals, a consolidant cannot alter stone properties such as porosity, color, gloss, etc. In conservation of granitic cultural heritage, polymeric consolidants composed by acrylic/methacrylic monomers, unsaturated polyesters, fluorinated polymers, epoxy resins, siloxanes, etc. have been commonly used but unfortunately many of these products modify the original properties of stones. Commonly, alkoxysilanes, such as tetraethoxysilane (TEOS) products are applied due to their ability to form siloxane bonds [19]. These products polymerize in situ inside the pore through a classic sol–gel process, with a significant increase of the cohesion of the material [19]. TEOS is composed by monomeric molecules that react with water to form polysiloxane [20]. It is classified as a silicon-based hybrid polymer since it fits into the definition of polymer by the International Union of Pure and Applied Chemistry (IUPAC) [21] (a substance composed of macromolecules of high relative molecular mass with a structure based on the repetition of units derived from molecules of low relative molecular mass known as monomers). In order to avoid the cracking registered in the TEOS coatings due to the capillary pressure in the gel inside the pore and the evaporation of the solvent upon the gelation process on the outer surface of the capillary network [22], recently, the addition of nanoscale SiO_2_ particles to polymers has been investigated [19,23]. They have become a suitable alternative because they allow a better safeguard of the stone properties and achieve higher penetration rates [19,23]. Usually, these nanoparticles are embedded in polymers [24,25,26]. Zendri et al. [24], applying nanoscale SiO_2_ particles with sodium silicate and ethyl silicate on calcium carbonate and quartz, found that this mixture constitutes a gel of amorphous silica after the evaporation of the solvent was transformed into xerogels. Moreover, organic–inorganic hybrids have been developed, where the hydrolysis is sufficiently rapid to provide hydrolyzed products that can condense with the polymer component [27,28,29,30]. Then, a homogeneous organic–inorganic hybrid gel is obtained and the cracking of the coatings is avoided. 

In order to create multifunctional products (consolidation and self-cleaning), the addition of nanocrystalline TiO_2_ to Si-based consolidants (ethyl silicate or nanosized silica) with particular attention given to the aesthetic effects on granite was investigated [31]. Tests of three different concentrations of TiO_2_ revealed that the lowest concentration (0.5 wt %) did not induce a visible color change on the granite surface, an advantage considering use of the consolidant as an intervention for the preservation of the built cultural heritage. Moreover, it was found that (1) higher penetration rates were identified in the granite coated with nanosized silica colloidal solution, while ethyl silicate was only found in the few first μm, and (2) the TiO_2_ addition seemed to reduce the penetration of the nanosized silica consolidant. However, in this previous study, the self-cleaning property achieved by the TiO_2_-spiked consolidants (with different composition) was not assessed. 

Therefore, as a follow-up study, the aim of the present research was to investigate the impact of composition of the abovementioned consolidants on the self-cleaning properties of the coatings spiked with different amounts of TiO_2_ (0.5, 1, and 3 wt %) applied to a granite commonly used in the architecture in the Northwest Iberian Peninsula. Moreover, untreated samples and samples coated with consolidant without TiO_2_ were included for comparative purposes. As previous research on self-cleaning evaluation of TiO_2-_spiked polymers, regardless of the substrate underneath, used synthesized substances such as Printex-U [11], orange dye [12], red dyes [13], etc. as proxies for real soot, it was necessary from the practical point of view to apply real soot from car engines. Moreover, in order to determine the effectiveness in enhancing the self-cleaning property of the different UV radiations, the traditionally used radiation on these types of studies, UV-A and novelty UV-B radiations, were tested. The self-cleaning efficacy of the treatments was monitored by conducting color measurements every 330 hours during 1650 hours (75 days). At the end of the experiment, the surfaces were inspected by stereomicroscopy and scanning electron microscopy. 

## 2. Materials and Methods 

### 2.1. Stone

**Figure 1:**
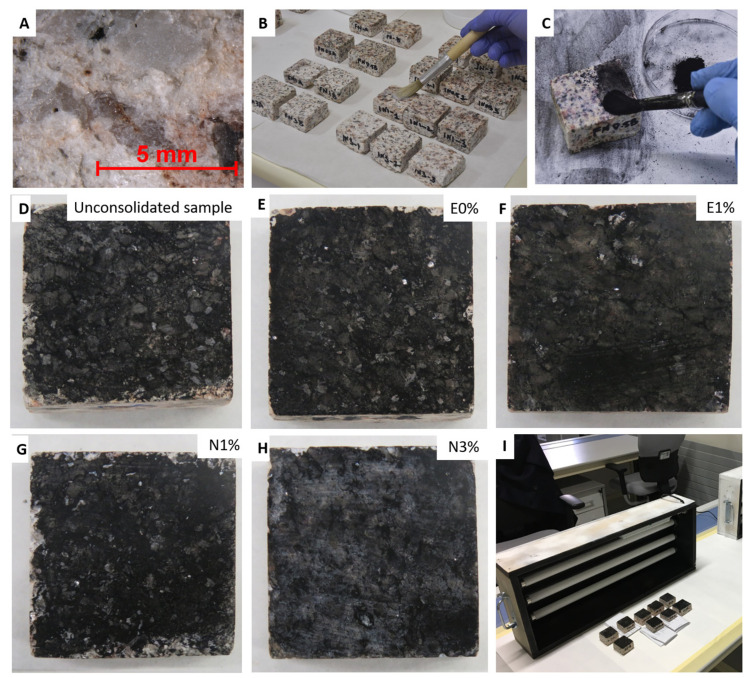
(**A**): Micrograph of the reference granite slab, taken with a stereomicroscope. (**B**): Photograph showing how the consolidant was applied. (**C**): Application of soot to a slab coated with the Nano Estel (N) containing 0.5% wt. TiO_2_. (**D**–**H**): Samples after being covered with the diesel soot. (**I**): Box with the UV-A radiation. See Table 1 for explanation of sample labeling.

The stone used in the study was a commercial granite, Rodas (Figure 1A,B), selected because it is commonly used in the architectonic heritage in the Northwest Iberian Peninsula. It is an alkaline-type granite containing 25% quartz, 31% albite, 26% microcline, 12% muscovite, 6% biotite, and accessory minerals such as apatite, zircon, rutile, sillimanite, chlorite, and opaque [32]. Its total porosity accessible to water is 6.50 ± 0.2% [33]. Fifty-five granite slabs of dimensions 4 cm x 4cm x 2cm with dish-cutting finish were used in the tests.

In order to create a physically damaged substrate in need of consolidation, the granite slabs were subjected to heat stress (500 °C) for 12 hours and then cooled with a jet of tap water. The slabs were then left undisturbed under laboratory conditions (15 ± 5 °C and 60 ± 10% Relative Humidity-RH) for two days. The whole process was repeated, thus increasing the total porosity accessible to water to 7.30 ± 0.4%, as previously described [33].

### 2.2. Consolidant Products

Two consolidant products with different compositions were tested following [31], an ethyl silicate consolidant and a consolidant based on nanosized silica. The layers of the consolidants were found to have a different texture, which may influence the self-cleaning capacity of TiO_2_-spiked consolidants. The consolidant products were provided by C.T.S. ESPAÑA (Getafe, Madrid, Spain) [34]:

- Estel 1000^®^ (hereinafter E) is composed of tetraethyl orthosilicate diluted in white spirit D40 (70 vol %). This is a ready-to-use colorless liquid, previously used in various scientific studies [3,35].
- Nano Estel^®^ (hereinafter N) is an aqueous colloidal solution of nanosized silica particles (10–30 nm). This product has been used in previous studies [3,36,37]. The nanosized silica particles bind to each other forming a silica xerogel, similar to that formed by ethyl silicate consolidants. The commercial product is concentrated and must be diluted with 1–2 parts of deionized water, as recommended by the supplier.

### 2.3. Sample Preparation

The titanium dioxide (TiO_2_) used as photocatalyst additive was commercial product, Aeroxide P-25, from Evonik Resource Efficiency GmbH (Barcelona, Spain) [38]. The product consists predominantly of nanocrystalline anatase (3/1, anatase/rutile ratio) with specific surface area of 50 m^2^ g^−1^ [39]. The TiO_2_ nanoparticles were rounded, hexagonally shaped, and of diameter of about 30 nm [39]. 

Table 1 shows the conditions and the codes used for each sample in the experiment. The commercial consolidants were directly mixed with TiO_2_ at three different concentrations (0.5, 1, and 3 wt %). Moreover, consolidant without TiO_2_ added (0 wt %) was also tested. Therefore, for each consolidant, four formulations were applied (each formulation was applied to six granite slabs). The consolidant products were applied by brush (Figure 1B) until the samples were saturated. Before application of the consolidant, as previously described [40], the stone surface was pretreated by a single application of ethanol, by brushing, in order to reduce the surface tension and thus enhance penetration of the product. The stone surface was then covered with Japanese craft paper. After four applications (with an interval of 48 hours between each), the slabs were maintained under laboratory conditions (18 ± 5 °C and 50 ± 10% RH), until they reached constant weight (approximately 30 days), in order to ensure polymerization of the products. 

Seven slabs without consolidant coatings were also included in the study for purposes of comparison; one slab was used as a reference sample and the other six slabs were used in the UV radiation tests. These untreated samples were maintained under laboratory conditions while the coated samples were being prepared.

After 30 days, samples were covered by soot collected from the exhaust pipes of diesel cars in the car park of the Mining and Energy Engineering School at the University of Vigo (Vigo, Spain). In order to facilitate adherence of the soot to the surface of the stone, the surfaces were wetted with a cotton swab before the soot was applied with a brush (Figure 1C) until the surfaces were completely black (Figure 1D–H).

### 2.4. UV Radiation Tests

Samples were exposed to one of two different types of UV radiation in closed boxes for 1650 h (75 days, with 2 hours off/day to avoid lamp overheating issues) (Figure 1I):

UV-A irradiation with three Philips (Koninklijke Philips N.V., Amsterdam, Netherlands) TL-D 18W (Actinic BL 5A Hg) lamps (spectral region 340–400 nm, main peak at 365 nm) 

UV-B irradiation with three Philips (Koninklijke Philips N.V., Amsterdam, Netherlands) Ultraviolet-B TL-200W/12RS lamps (spectral region 270–420 nm, main peak at 310 nm)

The same system of lamps was used in a previous study [41]. For each irradiation, three lamps were placed 2.5 cm above the samples and separated by 10 cm from each other. 

### 2.5. Analytical Techniques

The chemical composition of the diesel soot was characterized by x-ray fluorescence (XRF) using a Siemens SRS 3000 (Siemens AG, Berlin, Germany). This enabled the chemical composition of the major and trace elements to be determined. 

The soot was then applied to a glass slide for visualization by scanning electron microscopy (SEM) (Philips XL30, Koninklijke Philips N.V., Amsterdam, Netherlands) coupled with an energy dispersive X-ray spectrometry (EDS) (Oxford Inca Energy 300 SEM, Oxfordshire, UK) in backscattered electron (BSE) mode. Carbon-coated samples were visualized at an accelerating potential of 15–20 kV, a working distance of 9–11 mm, and specimen current of 60 mA. The acquisition time for recording EDS spectra, i.e., the dwell time, was 40–60 s.

In order to monitor the self-cleaning properties of the coatings, measured as the recovery of the initial color of the granite during the exposure of the samples to the UV radiation, the color of the surfaces was measured with a portable spectrophotometer (Minolta, model CM-700, KONICA MINOLTA, Chiyoda, Tokyo, Japan) equipped with CM-S100w (SpectraMagicTM NX, Chiyoda, Tokyo, Japan) software. Color was expressed in the CIELAB (Commission Internationale de l´Eclairage, CIE, Vienna, Austria) color space [42]. The following parameters were measured: lightness (L*), which varies from 0 (absolute black) to 100 (absolute white); a*, representing the redness–greenness range (+a*: red and −a*: green); b*, associated with yellowness–blueness spectrum (+b*: yellow and −b*: blue). Color measurements were made in specular component excluded (SCE) mode, for a spot diameter of 8 mm with diffuse illumination by means of xenon flash arc lamp and 10-nm diffuse bandwidth, using illuminant D65 at observer angle 10°. A total of 20 random readings were made on each surface, following [43]. For each sample, measurements were made on seven different occasions: (1) before being coated with the consolidant (unconsolidated granite slabs) or after being coated with the spiked consolidants (treated granite slabs); (2) three days after soot application; and (3) to (7) at intervals of 330 hours (15 days) during the UV radiation tests. The global color change (∆E*_ab_) was then computed as follows [42]:

(1) $$\Delta E_{ab}^{*}=\sqrt{{\left(\Delta L^{*}\right)}^{2}+{\left(\Delta a^{*}\right)}^{2}+{\left(\Delta b^{*}\right)}^{2}}$$

where

ΔL* = L*_p_ − L*_i_, (2)

Δa* = a*_p_ − a*_i_, (3)

Δb* = b*_p_ − b*_i_, (4)

and where i is the coordinate related to the initial color of the stone (unconsolidated or consolidated with/without nanocrystalline TiO_2_: first measurement) and p is the coordinate at the different measurement times previously indicated. 

Remains of diesel soot on the sample surface were detected by stereomicroscopy (Nikon SMZ645, Minato, Tokyo, Japan). Micrographs were taken of the uncoated reference slabs, coated slabs prior to soot application, and coated slabs before and after the UV radiation test.

The surfaces exposed to UV radiation (UV-A or UV-B) for 1650 h were evaluated using SEM-EDS (FEI- Thermo Fisher Scientific, Waltham, Massachusetts, United States- Quanta 200 in BSE mode). Carbon-sputtered surfaces were studied using the same optimum observations’ conditions used to characterize the soot. 

### 2.6. Statistical Analysis

Color data were subjected to one-way analysis of variance (ANOVA) and Tukey's Honestly-significant-difference (HSD) post hoc tests to compare the different treatments. Homogeneity of variance was tested using Levene's test, and the normality of residuals was checked using the Shapiro–Wilk test. Statistical significance was established as p < 0.05. Statistical tests were conducted using R software (R Development Core Team, Vienna, Austria) for MacOSX [44].

## 3. Results

The chemical composition of the diesel soot coincided with previously reported characterization data [45]. The major components were C (>94%) and S (4.26%) (Table 2). The other compounds were present at concentrations lower than 0.5%, with those of Si, P, Ca, Fe, Na, and Al being relatively highest.

**Figure 2:**
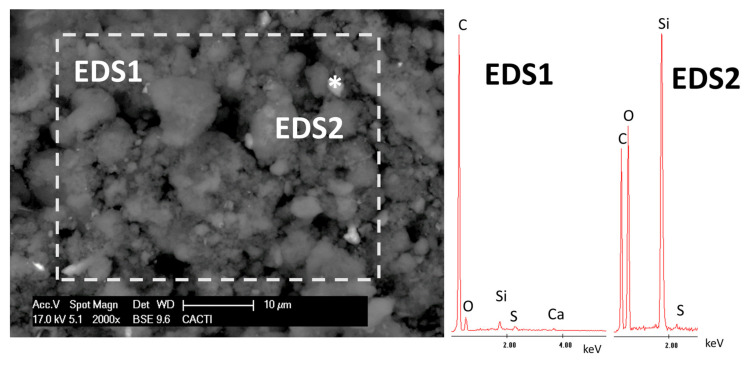
Micrograph of the diesel soot. The EDS spectra are also shown. * points out the area where the EDS2 was taken.

SEM observation of the diesel soot enabled identification of carbonaceous particles of different sizes mixed with Si-rich particles (Figure 2).

**Figure 3:**
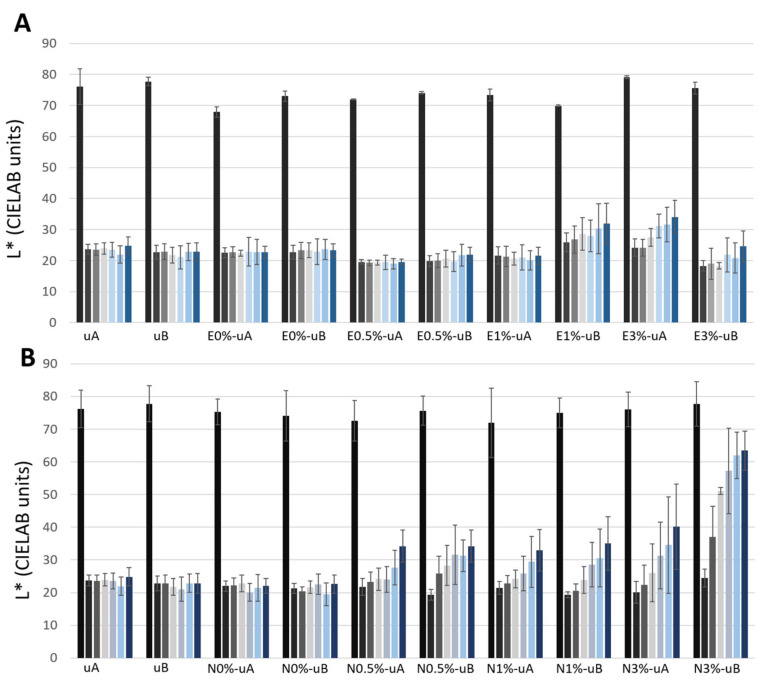
CIELAB parameter L* (lightness–darkness changes) of the samples during exposure for 1650 hours (75 days) to UV radiation (E-coated samples: (**A**) and N-coated samples: (**B**)). Each measurement was made at intervals of 330 hours (15 days), i.e., a total of seven measurements. The first bar of each group represents L* from the samples before being covered with soot, the second bar represents L* from the surface after soot application, and the other bars represent the measurements of the surfaces exposed to UV radiation every 330 hours (15 days). See Table 1 for sample labeling.

**Figure 4:**
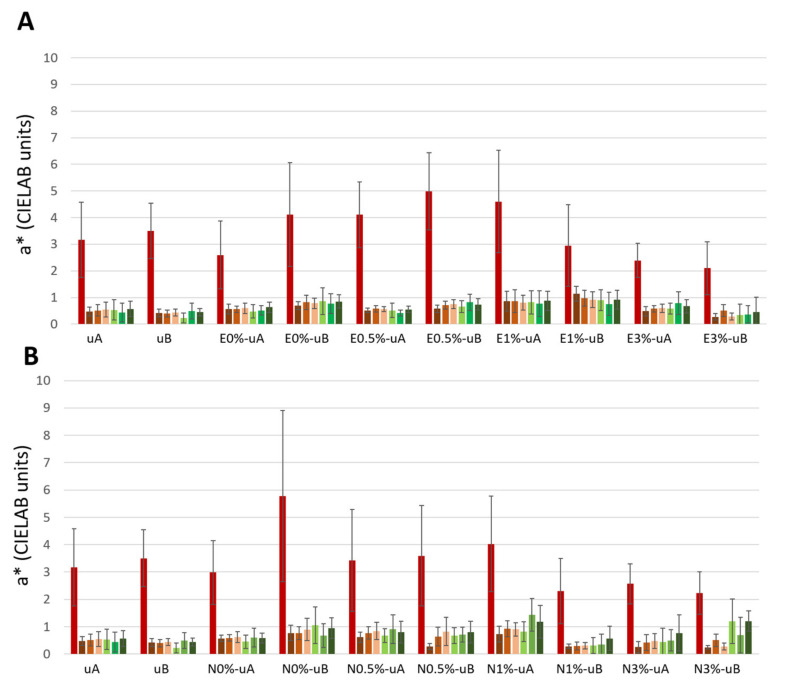
CIELAB parameter a* (redness–greenness changes) of the samples during exposure for 1650 hours (75 days) to UV radiation (E-coated samples: (**A**) and N-coated samples: (**B**)). Each measurement was made at intervals of 330 hours (15 days), i.e., a total of seven measurements. The first bar of each group represents a* from the samples before being covered with soot, the second bar represents a* from the surface after soot application, and the other bars represent the measurements of the surfaces exposed to UV radiation every 330 hours (15 days). See Table 1 for sample labeling.

**Figure 5:**
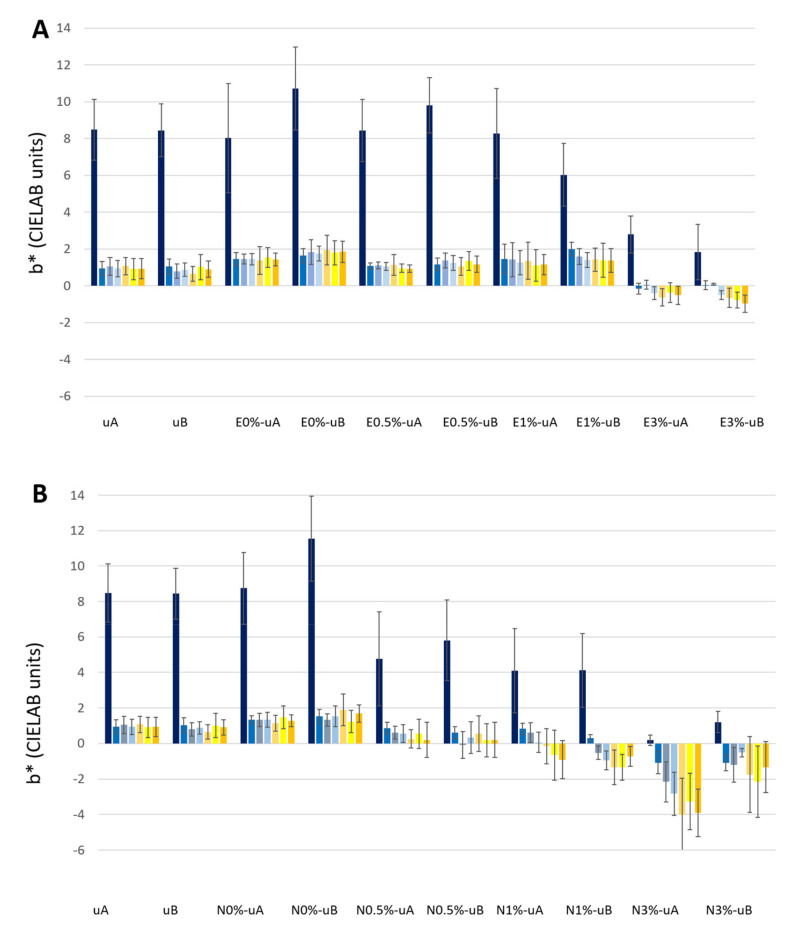
CIELAB parameter b* (yellowness–blueness changes) of the samples during exposure for 1650 hours (75 days) to UV radiation (E-coated samples: (**A**) and N-coated samples: (**B**)). Each measurement was made at intervals of 330 hours (15 days), i.e., a total of seven measurements. The first bar of each group represents b* from the samples before being covered with soot, the second bar represents b* from the surface after soot application, and the other bars represent the measurements of the surfaces exposed to UV radiation every 330 hours (15 days). See Table 1 for sample labeling.

Regarding the color parameters (Figure 3, Figure 4 and Figure 5, Table S1), the lightness (L*) parameter was the most affected by the application of the soot (Figure 3, Table S1), in all cases with decreases of more than 49 CIELAB units attributed to a notable darkening of the surface (compare Figure 1D–H and Figure 1A). The lowest ΔL* values (Table S1) were detected for E1%-uB (ΔE*_ab_ = 49.21 CIELAB units), N3%-uB (ΔE*_ab_ = 51.98 CIELAB units), and E3%-uA (ΔE*_ab_ = 51.37 CIELAB units). 

For the unconsolidated surfaces, L* (Figure 3, Table S1), a* (Figure 4, Table S1), and b* (Figure 5, Table S1) did not vary, regardless of the type of UV radiation (uA and uB samples). The same trend was also observed for the surfaces coated with both unmodified consolidants (without TiO_2_) (E0%-uA, E0%-uB, N0%-uA, and N0%-uB).

Regarding the samples with TiO_2_, different responses were identified considering the TiO_2_ concentrations and the consolidant used:

(1) For the surfaces coated with E spiked with 0.5% wt. TiO_2_ (Figure 3A), the slight changes in L* over time were not statistically significant differences for either type of radiation (0.5%-uA and 0.5%-uB). However, for the N-coated surfaces (Figure 3B), an increase in L* was already recorded on the 15th day of radiation exposure (third bar) relative to the measurement after the surface was covered with the soot (second bar of each group). Regarding the standard deviations, for N0.5%-uA, only the final measurement (seventh bar) was statistically significantly different from that obtained before the test (second bar), while for N0.5%-uB, the measurement on the 15th day (third bar) was statistically significantly different from that made on the surface only covered with soot (second bar).
(2) Regarding surfaces coated with 1% (wt.) TiO_2_-spiked consolidant, for the N consolidated surfaces, L* increased from the beginning of exposure (Figure 3B), yielding similar values of L* regardless of the radiation at the different times (15, 30, 45, 60, and 75 days). Considering the standard deviations, for the N1%-uA, the measurements were different (with statistically significant differences regarding the measurement of the surface just covered with soot, second bar) only after 75 days (seventh bar) and for the N1%-uB, after 45 days (fifth bar). For the E-coated surfaces (Figure 3A), two different responses were detected: for the surfaces irradiated with UV-A, there were no changes in L* over time, while for the surfaces irradiated with UV-B, the L* increased over time, although not statistically significantly different from the surfaces covered by soot (second bar).
(3) Evaluating the surfaces coated with formulations spiked with 3% wt. TiO_2_, for both consolidants, L* increased over time: the changes were more pronounced with the N consolidant, in the case of N3%-uB (Figure 3B) showing an almost complete recovery of the L* parameter (first bar); in fact, although the average L* was lower than the computed value for the uncoated surface (without soot, first bar), there were no statistically significant differences after the 45th day (fifth bar). Considering the E-coated surfaces, the L* was higher for the surfaces irradiated with UV-A than for those irradiated with UV-B. For the UV-A-irradiated surfaces (E3%-uA), comparing with the surface only coated with soot (second bar), only the L* for the surfaces after the 45th day (fifth bar) was statistically significant, while for the UV-B-irradiated surfaces (E3%-uB), there were no statistically significant differences at the different times. For the N samples (in contrast to the E-based samples), greater increases were observed in the UV-B-irradiated samples (N3%-uB) than in the UV-A samples (N3%-uA). For the N3%-uA, only the L* measurement of the surface at the end of the experiment (seventh bar) was statistically different from that measured on the surface only covered with soot (second bar). However, for the N3%-uB, statistically significant differences relative to the L* measured on the surface with the soot before the radiation exposure (second bar) were detected after the 30th day (fourth bar).

Regarding parameter a* (Figure 4, Table S1), after soot application, all the surfaces underwent a decrease in a* of between 1.78 to 4.33 CIELAB units, with the lowest value being recorded for E0%-uA and the highest for E1%-uA. 

In contrast to L*, a* did not show any general trend over time. Only a slight increase in a* values in N1% and N3% was observed, regardless of the type of UV radiation (N1%-uA, N1%-uB, N3%-uA, and N3%-uB, Figure 4B). Moreover, considering the standard deviations, the values obtained for the surfaces every 15 days were not statistically significantly different from those obtained for the surfaces only covered by the soot (second bar). 

The b* parameter decreased greatly after the soot was applied to the slabs, regardless of the consolidant coating on the surface (Figure 5, second bar, Table S1). The Δb* ranged between −6.08 CIELAB units (for both surfaces E0%-uB and E1%-uB) and −10.10 CIELAB unit (for N3%-uA).

In the granite slabs coated with TiO_2_-spiked consolidants, as reported for L*, different responses were obtained considering the consolidant composition and the TiO_2_ concentration:

(1) For the samples coated with E consolidant spiked with 0.5 wt % TiO_2_ (Figure 5A), no differences were found over time, regardless of the type of radiation (E0.5%-uA and E0.5%-uB), and the b* values were similar to those measured in the uncoated samples (uA and uB), and 0 wt % TiO_2_-spiked surfaces (E0%-uA and E0%-uB). However, for N, although the differences were not statistically significant, the b* parameter tended to decrease over time (Figure 5B).
(2) For the 1 wt % TiO_2_-spiked samples, two different trends were detected regarding the consolidant: (1) for the E-coated samples, b* decreased over time, although no statistically significant differences were detected relative to the surfaces covered with soot (Figure 5A, second bar) and (2) for the N-coated samples, b* ranged from positive values (ca. 0.5 CIELAB units) to negative values (Figure 5B). Relative to the measurement of the surface only covered with soot (second bar), for the UV-A-irradiated surfaces only the final measurement (N1%-uA, seventh bar) was statistically significantly different, while for the UV-B-irradiated samples (E1%-uA), already after the 30th day (fourth bar), the b* values were statistically different from the surface before the start of the test (second bar).
(3) For the samples coated with consolidant spiked with 3% TiO_2_-, the four conditions (Figure 5A, E3%-uA and E3%-uB; Figure 5B, N3%-uA and N3%-uB) showed decreases in b*, which reached negative values even after soot application before the beginning of the UV radiation tests. The changes in b* were greater in the N-coated samples (Figure 5B). The decreases in the E-coated samples (Figure 5A) were not statistically significant, relative to the color of the surface only covered with soot (second bar), except for the b* parameter measured on the last day for E3%-uB (Figure 5A, seventh bar). The differences were also not statistically different for N3%-uB, while in the counterpart irradiated with UV-A, the b* parameter decreased significantly since the 45th day (fifth bar).

**Figure 6:**
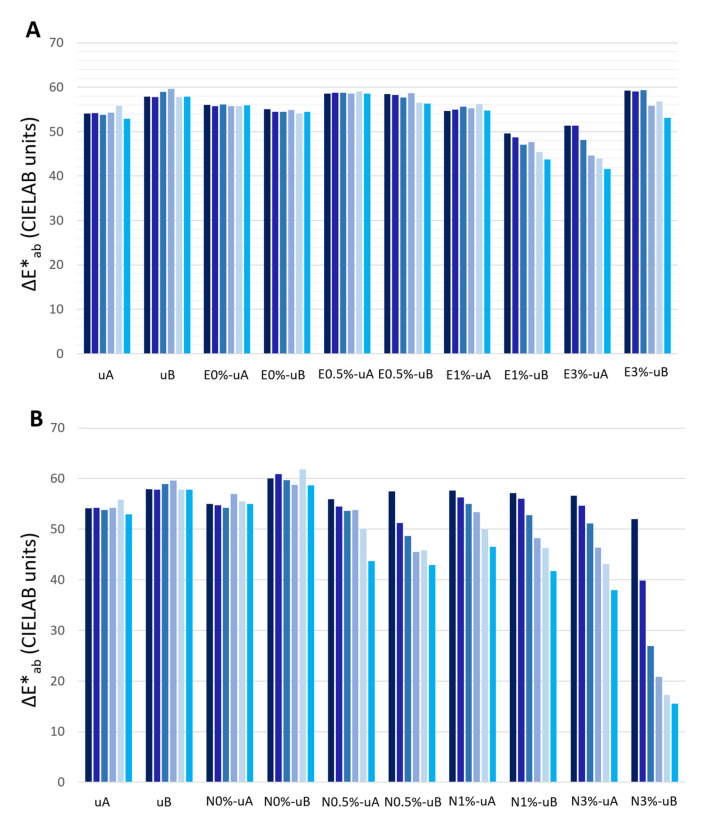
The ΔE*_ab_ (total color changes) of the samples during exposure for 1650 hours (75 days) to UV radiation (E-coated samples: (**A**) and N-coated samples: (**B**)). Each measurement was made every 330 hours (15 days), i.e., a total of six measurements, with the color of the unconsolidated surfaces or surface treated with the consolidant without soot as the reference. The first bar of each group represents the ΔE*_ab_ of the surface after soot application and the other bars represent the ΔE*_ab_ of the surfaces during exposure to UV irradiation every 330 hours (15 days). See Table 1 for sample labeling.

The ΔE*_ab_ values remained similar over time for the unconsolidated granite slabs (uA and uB) and for the surfaces coated with unmodified consolidant (without TiO_2_) and exposed to both types of radiation (E0%-uA, E0%-uB, N0%-uA, and N0%-uB), as expected considering that L*, a*, and b* did not vary over time (Figure 6). 

However, ΔE*_ab_ (reduction) was greater in the TiO_2_-spiked N slabs (Figure 6B) than in their E counterparts (Figure 6A). Indeed, for E0.5% slabs under both types of radiation (uA and uB) and E1% slabs under UV-A, ΔE*_ab_ was constant over time. It is important to highlight that the ΔE*_ab_ reduction rate did not increase over time in any samples, and even in N3%-uB this rate was reduced over time (Figure 6B). 

In the samples coated with the E-based consolidant (Figure 6A) containing 1% TiO_2_ and 3% TiO_2_, although for the E1%-uA, the ΔE*_ab_ did not vary over time, E1%-uB and both E3% samples (E3%-uA and E3%-uB) showed similar reductions in ΔE*_ab_ (ca. 9 CIELAB units). For the samples treated with the E-based consolidant containing 3% wt. TiO_2_, although higher ΔE*_ab_ values were obtained for the UV-B-radiated samples (E3%-uB) relative to the UV-A samples (E3%-uA), similar, decreasing trends were observed over time.

In the samples treated with the N-based consolidant (Figure 6B), in addition to the greater reduction in ΔE*_ab_ than those observed for the E-based samples, the ΔE*_ab_ reduction was greater in the samples irradiated with UV-B, particularly in the samples with higher TiO_2_ contents. In the final measurement (sixth bar), the ΔE*_ab_ decreased by 37.94 CIELAB units for the N3%-uA and 15.94 CIELAB units for the N3%-uB.

Considering that the ΔE*_ab_ values were computed considering as reference the color of the surfaces before soot application, all the changes were greater than 5 CIELAB units, i.e., the value below which a color change cannot be perceived by the human eye [46]. The lowest ΔE*_ab_ was 15.49 CIELAB units for the N3%-uB.

**Figure 7:**
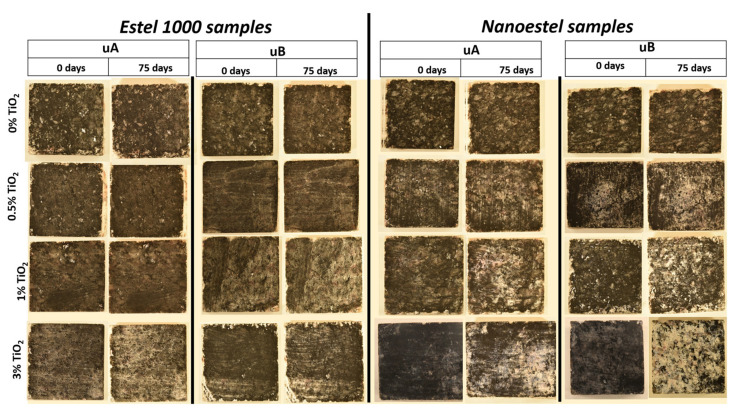
Digital photographs of the samples, before being exposed to UV radiation and after 1650 hours (75 days).

Figure 7 shows digital photographs of the samples before and after the UV irradiation tests. For the consolidated samples without TiO_2_, the samples did not show differences between the surface before starting the test and after the test. For the samples coated with TiO_2_-spiked consolidants, for E0.5%-uA, E0.5%-uB, and E1%-uA, no differences were observed before and after the test, while for the other conditions, a decrease in soot with different intensity was detected. This difference was greater for the surfaces with the highest TiO_2_ content tested (3%), specifically for the N-based samples (N3%-uA and N3%-uB).

**Figure 8:**
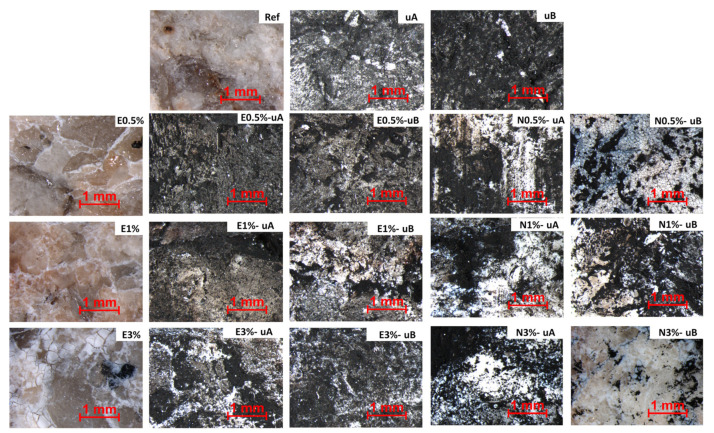
Micrographs of the uncoated samples before soot application (Ref) and after irradiation (uA and uB) and slabs coated with consolidants with different concentrations of TiO_2_, before the test (left column) and after UV (A or B) irradiation for 1650 h (75 days). See Table 1 for sample labeling.

These observations were confirmed by the stereomicroscopic findings (Figure 8). After the test, the surfaces of the uncoated, UV-irradiated slabs were totally covered by the soot. Although complete recovery of the surface color due to degradation of the soot was not observed on the slabs coated with TiO_2_-spiked consolidants, the appearance of the surface treated with the formulations with the highest TiO_2_ contents were more similar to the initial appearance. The best results in terms of soot reduction were obtained with the N-based formulations, as less soot was detected on the surfaces of the slabs coated with this consolidant after the test. 

Regarding the UV radiation, it was not possible to identify by simple visual examination which type of radiation yielded the best self-cleaning results, except for the slabs coated with the consolidant containing the highest TiO_2_ concentration. Thus, for the E-based formulations, the best cleaning results seemed to be obtained with UV-A while for the N-based formulations, UV-B yielded the highest rate of degradation of soot.

**Figure 9:**
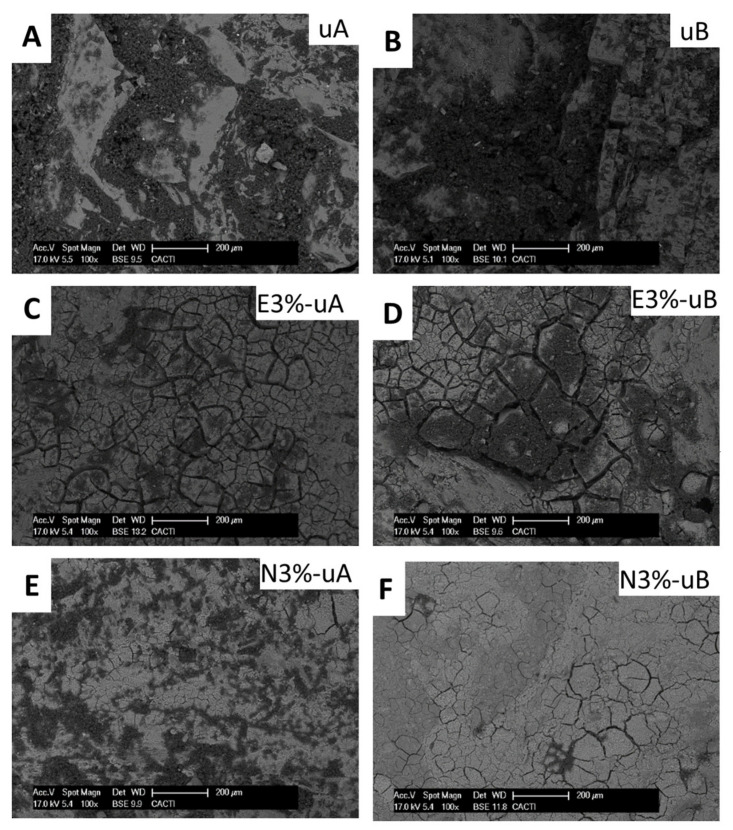
SEM micrographs of the uncoated samples after radiation tests: UV-A radiation (**A**) and UV-B radiation (**B**). SEM micrographs of the samples coated with the consolidants with the highest concentration of TiO_2_ (3 wt %) for E (**C** and **D**) and N (**E** and **F**). Samples shown in C and E were exposed to UV-A radiation and those shown in D and F were exposed to UV-B. See Table 1 for sample labeling.

SEM analysis enabled identification of soot on the surfaces and also the texture of the consolidant coatings. For the uncoated surfaces (Figure 9A,B) and the surfaces coated without TiO_2_, notable amounts of organic remains were detected on the surface and in cracks. The organic remains decreased with the TiO_2_ concentration (Figure 9C,D), confirming the spectrophotometric and stereomicroscopic findings. 

Regarding the surfaces coated with the formulations with the highest concentrations of TiO_2_ (Figure 9C–F), enhancement of the soot degradation was observed in those irradiated with UV-B (Figure 9D,F). 

Moreover, SEM enabled identification of different texture for both consolidant coatings (consolidant based on ethyl silicate: Figure 9C,D; consolidant based on nanosized silica: Figure 9E,F). The surfaces coated with E (Figure 9D) were less compact due to the existence of wider fissures than those in the surfaces coated with N (Figure 9F).

## 4. Discussion

The composition of the consolidant (an ethyl silicate and a colloidal solution of nanosized silica) mixed with the nanocrystalline TiO_2_ proved important in relation to the self-cleaning efficacy of the spiked products applied to highly weathered granite slabs. In the spectrophotometric monitoring of the soot degradation, lower color parameter changes (ΔL*, Δa*, Δb*, and ΔE*_ab_) indicated a higher degree of soot degradation, as the values were computed relative to the color of the surfaces before soot was applied. However, as none of the ΔE*_ab_ values were lower than 5 CIELAB units, i.e., the value below which a color change cannot be perceived by the human eye [46], the soot was not totally degraded, even in the conditions with the highest ΔE*_ab_ (N3%-uB). This was confirmed by stereomicroscopic and SEM analysis, which detected carbonaceous particles on the sample surfaces. In a previous study with the same products and the same concentrations of TiO_2_, the impact of TiO_2_-spiked consolidants on the appearance of granite was evaluated [31]. The lowest TiO_2_ concentration tested (0.5 wt %), regardless of the consolidant, yielded ΔE*_ab_ lower than 5 CIELAB units, which is also associated with a low to medium risk of being incompatible with conservation purposes [47]. In the present study, recovery of the initial color of the granite was higher in the samples coated with the consolidant containing nanosized silica than in the samples coated with the ethyl silicate-based consolidant. The colloidal solution of nanosized silica thus displayed the highest self-cleaning efficacy, due to intense photocatalytic activity. On the surfaces coated with nanosized colloidal silica, SEM analysis detected a more compact coating with narrower fissures, while in the ethyl silicate-based coatings, the fissures were wider, as previously reported [31]. As a consequence of the ethyl silicate-based layers being less compact, the treated surfaces had fewer TiO_2_ nanoparticles available to be activated under light irradiation and, therefore, fewer electrons and positive holes were generated in the conduction and valence band of TiO_2_ [48]. The holes can either react directly with organic molecules or form hydroxyl radicals that oxidize organic molecules [49]. The electrons can also react with organic compounds to yield reduction products. 

Regarding the color parameters measured (L*, a*, and b*), L* (mainly) and b* were the parameters most affected by soot degradation, as reported in other studies involving the self-cleaning efficacy of spiked polymers applied to carbonate stone, such as limestone and marble [3,5]. In a study involving mortars, ΔL* and Δb* were reported to represent accumulation of dirt on the stone surface [50]. The increased L* observed in the current research indicates the degree of self-cleaning over time. For E3%, N1%, and N3% (the highest TiO_2_ concentrations), the b* changed from positive values (+b*) to negative values (−b*), i.e., the color tended to become bluish over time, indicating the cleaning efficacy of the TiO_2_. However, for the other conditions, although the b* decreased over time, it remained positive (+b*). Positive values of b* are consistent with the yellowing associated with soot deposition [5], which has been ascribed to the mineralization of organic compounds from the soot adsorbed on the surface [50]. Therefore, −b* values correspond to the most effective conditions for the removal of yellowing, soiling deposit.

Considering the TiO_2_ content, for the surfaces coated with the lowest concentration of TiO_2_, different effects were observed for both consolidants. (1) For the samples coated with the ethyl silicate consolidant, the lowest TiO_2_ concentration did not induce self-cleaning and (2) for the samples coated with the nanosized silica-based consolidant, the lowest TiO_2_ concentration (0.5 wt %), regardless of the type of UV radiation, self-cleaning occurred, as indicated by the decrease in ΔE*_ab_. The soot degradation increased with the TiO_2_ concentration in the mixture. Regardless of the consolidant used, the mixtures containing 0.5% wt. TiO_2_ yielded ΔE*_ab_ below 5 CIELAB units [29], and their application thus prevented visible effects on the original color of the granite. Conversely, products containing 1 and 3 wt % TiO_2_ produced visible color changes. In addition, 1 and 3 wt % TiO_2_ also represent a medium to high risk of incompatibility with conservation of cultural heritage objects, because the associated ΔE*_ab_ is higher than 5 CIELAB units [45]. The deontological code for cultural heritage conservation expressed in the Venice charter states that a conservation treatment should not modify the properties of the treated materials, such as the color and brightness [51]. Thus, and considering that the mixtures containing ethyl silicate and the lower TiO_2_ concentrations did not have self-cleaning properties, the colloidal solution of nanosized silica containing 0.5 wt % TiO_2_ produced the most promising results. However, if the absence of color change is not important, coatings containing higher concentrations of TiO_2_ could be used due to the more effective self-cleaning properties.

Moreover, regardless of the consolidant used, soot degradation remained almost constant over time, except for E0.5% (uA and uB), which did not have self-cleaning, and N3%-uB, which showed a soot degradation rate decreased over time. The decrease in photocatalytic activity can be attributed to the aging-related modification of the consolidant, altering its capacity to retain the nanoparticles on the surface and the consequent loss of TiO_2_ nanoparticles [5]. However, as the experiments in the present study were performed in closed boxes, the samples were not subjected to washing by water from precipitation. Thus, the decrease in photocatalytic activity was not as intense as reported in the aforementioned study [5]. The photocatalytic activity decreases due to the deactivation of photocatalysis when TiO_2_ nanoparticles adsorb ions on their surface [52]. As a consequence, washing or rinsing the surface after a certain time is recommended to recover the photocatalytic activity by reactivating the TiO_2_ sites [5,50].

Regarding the influence of the UV radiation on the self-cleaning achieved by the spiked consolidants, UV-B radiation was the most effective in terms of soot degradation, except on the surface coated with the ethyl silicate and 3 wt % TiO_2_ (E3%), with which UV-A achieved better results in terms of soot degradation. Photogeneration of electron-hole pairs occurs when the energy of the incident photon is greater than the band gap of the semiconductor material. In low band gap materials, such as PbS (0.37 eV) and GaAs (1.43 eV), solar energy is sufficient to activate the process. By contrast, in materials with a high band gap, UV radiation is needed for activation. The typical band-gap energy of TiO_2_ is between 3–3.2 eV. Thus, at least UV-A radiation (wavelength < 400 nm) is needed for its activation [53], and better results are achieved with shorter wavelength radiation (more energetic according to Planck's theory [41]) such as UV-B (wavelength between 315 nm and 280 nm). Thus, poorer performance is expected when longer wavelength light is applied and vice versa, i.e., the self-cleaning results of TiO_2_ are maximized when activated by UV-B radiation. The different performance of E3% may be related to the interaction between the TiO_2_ nanoparticles (3 wt %) and the ethyl silicate. One of the limitations of TiO_2_ photocatalysis is the agglomeration of TiO2 nanoparticles due to their bonding by the organic ionizable molecules [54,55] from the ethyl silicate. Such agglomeration may prevent the active centers from receiving the light radiation and consequently hamper the photocatalytic activity of TiO_2_ [56].

## 5. Conclusions

This study evaluated how the self-cleaning properties of different consolidants were affected by composition and by the addition of different concentrations of nanocrystalline TiO_2_. Two consolidants were selected for study: (1) an ethyl silicate and (2) a nanosized silica-based solution, which were combined with three different concentrations (0.5, 1, and 3 wt %) of nanocrystalline TiO_2_. Granite slabs coated with the different formulations were exposed to different types of UV radiation (UV-A and novelty UV-B) for 1650 h in order to evaluate the effect of the TiO_2_ concentration on the self-cleaning property and to determine the most effective type of UV radiation. Degradation of real soot deposited on the surfaces was monitored using spectrophotometry and the final surfaces were evaluated by stereomicroscopy and SEM.

Although some degree of self-cleaning was detected in all samples, except the sample coated with the ethyl silicate-based consolidant containing 0.5 wt % TiO_2_, the degree of self-cleaning depended on the consolidant composition and the subsequent micro-texture of the coating, the TiO_2_ content, and the type of UV radiation applied. 

Although the soot was not completely degraded and the self-cleaning efficacy increased with the TiO_2_ concentration, self-cleaning was more efficient on the slabs coated with the nanosized silica-based consolidant, because more TiO_2_ nanoparticles were available to be activated by the radiation. The coatings containing this consolidant were more compact with narrower fissures than the ethyl silicate-based coatings. 

Regardless of the consolidant product, soot degradation rate over time remained constant or decreased over time. UV-B radiation achieved higher soot degradation rates than UV-A radiation. 

The TiO_2_-spiked, nanosized silica-based consolidant mixture with the lowest TiO_2_ (0.5 wt %) content could potentially be applied to granite in cultural heritage because (1) the coating did not induce a visible color change, (2) the higher the TiO_2_ concentration, the more intense the reduction on the consolidant effect [31], and (3) the ethyl silicate consolidant spiked with the lowest concentration TiO_2_ content did not induce self-cleaning. Further studies should be conducted to evaluate the performance of the selected mixture exposed to outdoor conditions.

## Figures and Tables

**Table 1:** Samples used in the experiment. For each condition, n = 3.

**TiO_2_ (wt %)**	**Without Consolidant**			
	**Exposure to UV-A**		**Exposure to UV-B**	
0	uA		uB	
**TiO_2_ (wt %)**	**With Consolidant**			
	**Estel1000**		**Nano Estel**	
	**UV-A**	**UV-B**	**UV-A**	**UV-B**
0	E0%-uA	E0%-uB	N0%-uA	N0%-uB
0.5	E0.5%-uA	E0.5%-uB	N0.5%-uA	N0.5%-uB
1	E1%-uA	E1%-uB	N1%-uA	N1%-uB
3	E3%-uA	E3%-uB	N3%-uA	N3%-uB

**Table 2:** Chemical composition of diesel soot used in this study, determined by x-ray fluorescence (XRF) analysis.

Compound	Content (%)
C	94.04
Na	0.17
Mg	0.05
Al	0.15
Si	0.374
P	0.283
S	4.26
Cl	0.026
K	0.0454
Ca	0.205
Ti	0.0394
Cr	0.0678
Mn	0.0015
Fe	0.2
Ni	0.002
Cu	0.0415
Zn	0.0295
Br	0.00056
Sr	0.00069
Ba	0.0035

## Supplementary Materials

The following are available online at https://www.mdpi.com/2073-4360/12/11/2577/s1. Table S1: Color parameters (L*, a*, and b*) and their respective variations during their exposure (15, 30, 45, 60, and 70 days) to the different UV radiations (UV-A: uA and UV-B: uB) considering the uncoated surfaces or those with consolidants (Nano Estel-N and Estel1000-E) and different TiO_2_ contents (0, 0.5, 1, and 3%TiO_2_) as the reference color (i.e., ref in the ID of each sample). Moreover, the color changes of the surfaces after being covered with soot were also determined. Check Table 1 for the labeling. Moreover, the global color change (ΔE*_ab_) was also computed; *n* = 60.

## References

[1] EC, European Commission. Energy and Smart cities – European Commission’s priority policies. https://ec.europa.eu/info/eu-regional-and-urban-development/topics/cities-and-urban-development/city-initiatives/smart-cities_en.

[2] Quagliarini E., Bondioli F., Battista Goffredo G., Cordoni C., Munafo P. (2012). Self-cleaning and de-polluting stone surfaces: TiO_2_ nanoparticles for limestone. Constr. Build. Mater..

[3] La Russa M.F., Ruffolo S.A., Rovella N., Belfiore C.M., Palermo A.M., Guzzi M.T., Crisci G.M. (2012). Multifunctional TiO_2_ coatings for Cultural Heritage. Prog. Org. Coat..

[4] Gherardi F., Colombo A., D’Arienzo M., Di Credico B., Goidanich S., Morazzoni F., Simonutti R., Toniolo L. (2016). Efficient self-cleaning treatments for built heritage based on highly photo-active and well-dispersible TiO_2_ nanocrystals. Microchem. J..

[5] Colangiuli D., Lettieri M., Calia A. (2019). Field study in an urban environment of simultaneous self-cleaning and hydrophobic nanosized TiO_2_-based coatings on stone for the protection of building surface. Sci. Total Environ..

[6] Kardar P., Amini R. (2019). Self-cleaning treatment on historical stone surface via titanium dioxide nanocoatings. Pigment. Resin Technol..

[7] Rivas T., Pozo S., Paz M. (2014). Sulphur and oxygen isotope analysis to identify sources of sulphur in gypsum-rich black crusts developed on granites. Sci. Total Environ..

[8] Comite V., Pozo-Antonio J.S., Cardell C., Randazzo C., La Russa M.F., Fermo P. (2020). A multi-analytical approach for the characterization of black crusts on the facade of an historical cathedral. Microchem. J..

[9] Ortega-Morales B.O., Reyes-Estebanez M.M., Gaylarde C.C., Camacho-Chab J.C., Sanmartín P., Chan-Bacab M.J., Granados-Echegoyen C.A., Pereañez-Sacarias J.E. (2018). Antimicrobial Properties of Nanomaterials Used to Control Microbial Colonization of Stone Substrata. Advanced Materials for the Conservation of Stone.

[10] Pozo-Antonio J.S., Rivas T., Fiorucci M.P., Ramil A., López A.J. (2016). Effectiveness of granite cleaning procedures in cultural heritage: A review. Sci. Total Environ..

[11] Smits M., Kit Chan C., Tytgat T., Craeye B., Costarramone N., Lacombe S., Lenaerts S. (2013). Photocatalytic degradation of soot deposition: Self-cleaning effect on titanium dioxide coated cementitious materials. Chem. Eng. J..

[12] Vasconcelos G., Carneiro J., Fernandes F., Jesus C., Palha C. Experimental analysis on the functional properties of rendering mortars with superficial addition of TiO_2_ nanoparticles. Proceedings of the 9 International Masonry Conference.

[13] Cohen J.D., Sierra-Gallego G., Tobón J.I. (2015). Evaluation of photocatalytic properties of Portland cement blended with titanium oxynitride (TiO_2__xNy). Nanopart. Coat..

[14] Bengtsson N., Castellote M. (2014). Heterogeneous photocatalysis on construction materials: Effect of catalyst properties on the efficiency for degrading NOx and self-cleaning. Mater. Constr..

[15] Luttrell T., Halpegamage S., Tao J., Kramer A., Sutter E., Batzill M. (2014). Why is anatase a better photocatalyst than rutile?—Model studies on epitaxial TiO_2_ films. Sci. Rep..

[16] Zhang J., Zhou P., Liu J., Yu J. (2014). New understanding of the difference of photocatalytic activity among anatase, rutile and brookite TiO_2_. J. Phys. Chem. Chem. Phys..

[17] Akakuru O.U., Iqbal Z.M., Wu A., Wu A., Ren W. (2020). TiO2 Nanoparticles: Properties and Applications. TiO_2_ Nanoparticles: Applications in Nanobiotechnology and Nanomedicine.

[18] McKenzie R.L., Aucamp P.J., Bais A.F., Bjorn L.O., Ilyas M., Madronich S. (2011). Ozone depletion and climate change: Impacts on UV radiation. Photochem. Photobiol. Sci..

[19] Wheeler G. (2005). Alkoxysilanes and the Consolidation of Stone.

[20] Sierra-Fernandez A., Gomez-Villalba L.S., Rabanal M.E., Fort R. (2017). New nanomaterials for applications in conservation and restoration of stony materials: A review. Materiales de Construcción.

[21] IUPAC. https://iupac.org/polymer-edu/what-are-polymers/.

[22] Mosquera M.J., Pozo J., Esquivias L. (2003). Stress during Drying of Two Stone Consolidants Applied in Monumental Conservation. J. Sol. Gel Sci. Technol..

[23] De Rosario I., Elhaddad F., Pan A., Benavides R., Rivas T., Mosquera M.J. (2015). Effectiveness of a novel consolidant on granite: Laboratory and in situ results. Constr. Build. Mater..

[24] Zendri E., Biscontin G., Nardini I., Riato S. (2007). Characterization and reactivity of silicatic consolidants. Constr. Build. Mater..

[25] Esposito Corcione C., Striani R., Frigione M. (2014). Organic-Inorganic UV-cured methacrylic-based hybrids as protective coatings for different substrates. Prog. Org. Coat..

[26] Frigione M., Lettieri M. (2018). Novel attribute of organic-inorganic hybrid coatings for protection and preservation of materials (stone and wood) belonging to Cultural Heritage. Coatings.

[27] Mascia L., Prezzi L., Haworth B. (2006). Substantiating the role of phase bicontinuityand interfacial bonding in epoxy-silica nanocomposites. J. Mater. Sci..

[28] Mosquera M.J., Santos D.M.D.L., Rivas T., Sanmartín P., Silva B. (2009). New Nanomaterials for Protecting and Consolidating Stone. J. Nano Res..

[29] Corcione C.E., Frigione M. (2013). Surface characterization of novel hydrophobic UV-curable siloxane-modified methacrylate/boehmite nanocomposites. Polym. Compos..

[30] Lionetto F., Mascia L., Frigione M. (2019). Evolution of transient states and properties of an epoxy-silica hybrid cured at ambient temperature. Eur. Polym. J..

[31] Pozo-Antonio J.S., Noya D., Montojo C. (2020). Aesthetic Effects on Granite of Adding Nanoparticle TiO_2_ to Si-Based Consolidants (Ethyl Silicate or Nano-Sized Silica). Coatings.

[32] IGME (Instituto Geológico y Minero de España) (1981). Mapa geológico de España. Serie Magna. E 1:50000.

[33] RILEM Recommandations provisoires, Essais recommandés pour mesurer l’altération des pièrres. Test n. II.1 Open Porosity, Commission 25 PEM, Protection et Erosion des Monuments. 1980.

[34] C.T.S.; C.T.S. España. https://www.ctseurope.com/es/.

[35] Cultrone G., Ibáñez V.M.S. (2018). Consolidation with ethyl silicate: How the amount of product alters the physical properties of the bricks and effects their durability. Materiales de Construcción.

[36] Zornoza-Indarta A., Lopez-Arce P. (2016). Silica nanoparticles (SiO_2_): Influence of relative humidity in stone consolidation. J. Cult. Herit..

[37] Pozo-Antonio J.S., Otero J., Alonso P., Mas i Barberà X. (2019). Nanolime- and nanosilica-based consolidants applied on heated granite and limestone: Effectiveness and durability. Constr. Build. Mater..

[38] EVONIK. https://www.corporate.evonik.com/.

[39] Pozo-Antonio J.S., Dionísio A. (2017). Physical-mechanical properties of mortars with addition of TiO_2_ nanoparticles. Constr. Build. Mater..

[40] Falchi L., Balliana E., Izzo F., Agostinetto L., Zendri E. (2013). Distribution of nanosilica dispersions in Lecce stone. Sci. Ca´Foscari.

[41] Pozo-Antonio J.S., Sanmartín P. (2018). Exposure to artificial daylight or UV-irradiation (A, B or C) prior to chemical cleaning: An effective combination for removing phototrophs from granite. Biofouling.

[42] CIE S014-4/E: 2007 (2007). Colorimetry Part 4: CIE 1976 L*a*b* Colour Space.

[43] Prieto B., Sanmartín P., Silva B., Martinez-Verdú F. (2010). Measuring the color of granite rocks. A proposed procedure. Color. Res. Appl..

[44] R Core Team R: A Language and Environment for Statistical Computing; R Foundation for Statistical Computing. http://www.R-project.org/.

[45] Khobragade R., Singh S.K., Shukla P.C., Gupta T., Al-Fatesh A.S., Agarwal A.K., Labhasetwar N.K. (2019). Chemical composition of diesel particulate matter and its control. Catal. Rev..

[46] García O., Malaga K. (2012). Definition of the procedure to determine the suitability and durability of an anti-graffiti product for application on cultural heritage porous materials. J. Cult. Herit..

[47] Rodrigues J.D., Grossi A. (2007). Indicators and ratings for the compatibility assessment of conservation actions. J. Cult. Herit..

[48] Poulopoulos S., Philippopoulos C. (2004). Photo-assisted oxidation of chlorophenols in aqueous solutions using hydrogen peroxide and titanium dioxide. J. Environ. Sci. Health.

[49] Ahmad R., Ahmad Z., Khan A.U., Mastoi N.R., Aslam M., Kim J. (2016). Photocatalytic systems as an advanced environmental remediation: Recent developments, limitations and new avenues for applications. J. Environ. Chem. Eng..

[50] Diamanti M.V., Paolini R., Rossini M., Basak Aslan A., Zinzi M., Poli T., Pedeferri M.P. (2015). Long term self-cleaning and photocatalytic performance of anatase added mortars exposed to the urban environment. Constr. Build. Mater..

[51] Venice Charter. The Venice Charter for the Conservation and Restoration of Monuments and Sites, 1964. http://www.icomos.org/venicecharter2004/index.html.

[52] Guillard C., Puzenat E., Lachheb H., Houas A., Herrmann J.M. (2005). Why inorganic salts decrease the TiO_2_ photocatalytic efficiency. Int. J. Photoenergy.

[53] Gomes J., Lincho J., Domingues E., Quinta-Ferreira R.M., Martins R.C. (2019). N–TiO_2_ photocatalysts: A review of their characteristics and capacity for emerging contaminants removal. Water.

[54] Chen D., Cheng Zhou Y., Chen N.P., Wang Y., Li K., Huo S., Cheng P., Peng P., Zhang R., Wang L. (2020). Photocatalytic degradation of organic pollutants using TiO_2_-based photocatalysts: A review. J. Cleaner Prod..

[55] Cervantes-Avilés P., Piñas N.C., Ida J., Cuevas-Rodríguez G. (2017). Influence of wastewater type on the impact generated by TiO_2_ nanoparticles on the oxygen uptake rate in activated sludge process. J. Environ. Manag..

[56] Mallakpour S., Nikkhoo E. (2014). Surface modification of nano-TiO_2_ with trimellitylimido-amino acid-based diacids for preventing aggregation of nanoparticles. J. Adv. Powder Technol..

