# Supplementary material for: Moving toward Smart Cities: Evaluation of the Self-Cleaning Properties of Si-Based Consolidants Containing Nanocrystalline TiO2 Activated by Either UV-A or UV-B Radiation
Source: Polymers (Basel). 2020 Nov 2;12(11):2577. doi: 10.3390/polym12112577 (PMC7692194; doi:10.3390/polym12112577)
Supplement: Supplementary file 1 [file polymers-12-02577-s001.pdf]

Table S1: Colour parameters (L*, a* and b*) and their respective variations during their exposure (15, 30, 45, 60 and 70 days) to the different UV-radiations (UV-A: uA and UV-B: uB) considering the uncoated surfaces or those with consolidants (NanoEstel-N and Estel1000-E) and different TiO_2 contents (0, 0.5, 1 and 3%TiO_2) as the reference color (i.e. ref in the ID of each sample). Moreover, the color changes of the surfaces after being covered with soot was also determined. Check Table 1 for the labelling. Moreover, the global color change (ΔE^*_{ab}) was also computed. n=60.

ID	L*	a*	b*	ΔL*	Δa	Δb*	ΔE^*_{ab}
uA-ref	77.269	3.11	7.72				
uA-soot	23.65	0.48	0.95	−53.62	−2.63	−6.77	54.11
uA-15	23.56	0.52	1.05	−53.71	−2.59	−6.67	54.18
uA-30	23.93	0.55	0.93	−53.34	−2.56	−6.79	53.83
uA-45	23.51	0.54	1.08	−53.76	−2.57	−6.64	54.23
uA-60	21.94	0.43	0.91	−55.33	−2.67	−6.81	55.81
uA-75	24.84	0.57	0.93	−52.43	−2.53	−6.79	52.93
uB-ref	80.13	2.94	8.08				
uB-soot	22.76	0.42	1.04	−57.38	−2.52	−7.04	57.86
uB-15	22.83	0.40	0.80	−57.30	−2.54	−7.28	57.82
uB-30	21.70	0.44	0.88	−58.43	−2.50	−7.20	58.93
uB-45	21.05	0.22	0.64	−59.08	−2.71	−7.44	59.61
uB-60	22.82	0.49	1.02	−57.31	−2.44	−7.06	57.80
uB-75	22.79	0.45	0.90	−57.34	−2.49	−7.17	57.84
E0%-uA-ref	78.12	2.35	7.91				
E0%-uA-soot	22.52	0.57	1.46	−55.60	−1.78	−6.45	56.00
E0%-uA-15	22.75	0.56	1.45	−55.38	−1.79	−6.46	55.78
E0%-uA-30	22.36	0.59	1.45	−55.76	−1.76	−6.46	56.16
E0%-uA-45	22.85	0.48	1.39	−55.27	−1.87	−6.52	55.69
E0%-uA-60	22.76	0.52	1.53	−55.37	−1.84	−6.38	55.76
E0%-uA-75	22.63	0.63	1.43	−55.49	−1.72	−6.48	55.89
E0%-uB-ref	77.37	2.69	7.71				
E0%-uB-soot	22.70	0.70	1.64	−54.67	−1.99	−6.08	55.04
E0%-uB-15	23.28	0.82	1.83	−54.09	−1.87	−5.89	54.44
E0%-uB-30	23.30	0.78	1.75	−54.07	−1.91	−5.97	54.43
E0%-uB-45	22.85	0.86	1.94	−54.51	−1.83	−5.77	54.85
E0%-uB-60	23.60	0.77	1.80	−53.76	−1.91	−5.92	54.12
E0%-uB-75	23.27	0.85	1.85	−54.10	−1.84	−5.86	54.45
E0.5%-uA-ref	77.48	3.40	8.96				
E0.5%-uA-soot	19.54	0.52	1.08	−57.94	−2.88	−7.88	58.55
E0.5%-uA-15	19.28	0.58	1.11	−58.19	−2.83	−7.86	58.79
E0.5%-uA-30	19.30	0.56	1.05	−58.18	−2.84	−7.91	58.78
E0.5%-uA-45	19.50	0.51	1.14	−57.98	−2.89	−7.83	58.58
E0.5%-uA-60	19.02	0.42	0.96	−58.46	−2.98	−8.00	59.08
E0.5%-uA-75	19.58	0.55	0.93	−57.90	−2.85	−8.03	58.52
E0.5%-uB-ref	77.86	3.14	7.75				

E0.5%-uB-soot	19.84	0.58	1.17	−58.03	−2.56	−6.58	58.46
E0.5%-uB-15	19.98	0.71	1.37	−57.88	−2.42	−6.38	58.28
E0.5%-uB-30	20.60	0.75	1.25	−57.27	−2.38	−6.51	57.68
E0.5%-uB-45	19.63	0.66	1.05	−58.24	−2.48	−6.70	58.68
E0.5%-uB-60	21.76	0.82	1.34	−56.11	−2.32	−6.42	56.52
E0.5%-uB-75	21.95	0.74	1.17	−55.91	−2.40	−6.58	56.35
E1%-uA-ref	75.44	5.19	9.78				
E1%-uA-soot	21.61	0.86	1.45	−53.82	−4.33	−8.33	54.64
E1%-uA-15	21.31	0.86	1.42	−54.13	−4.32	−8.36	54.94
E1%-uA-30	20.59	0.81	1.26	−54.85	−4.38	−8.52	55.68
E1%-uA-45	20.98	0.82	1.36	−54.46	−4.37	−8.42	55.28
E1%-uA-60	20.07	0.77	1.10	−55.37	−4.41	−8.68	56.21
E1%-uA-75	21.52	0.87	1.15	−53.92	−4.31	−8.63	54.78
E1%-uB-ref	75.08	3.25	8.08				
E1%-uB-soot	25.87	1.14	2.00	−49.21	−2.11	−6.08	49.63
E1%-uB-15	26.81	0.97	1.60	−48.27	−2.27	−6.47	48.76
E1%-uB-30	28.58	0.92	1.40	−46.51	−2.33	−6.67	47.04
E1%-uB-45	27.97	0.90	1.42	−47.11	−2.34	−6.66	47.64
E1%-uB-60	30.28	0.76	1.39	−44.80	−2.49	−6.69	45.37
E1%-uB-75	31.88	0.92	1.38	−43.20	−2.33	−6.70	43.78
E3%-uA-ref	74.81	3.27	7.80				
E3%-uA-soot	24.14	0.49	−0.15	−50.68	−2.79	−7.96	51.37
E3%-uA-15	24.11	0.58	0.06	−50.70	−2.69	−7.74	51.36
E3%-uA-30	27.48	0.60	−0.40	−47.34	−2.67	−8.20	48.12
E3%-uA-45	31.08	0.58	−0.63	−43.73	−2.69	−8.43	44.62
E3%-uA-60	31.67	0.79	−0.36	−43.14	−2.48	−8.17	43.98
E3%-uA-75	34.09	0.68	−0.51	−40.73	−2.60	−8.32	41.65
E3%-uB-ref	77.14	2.32	6.93				
E3%-uB-soot	18.29	0.27	0.04	−58.85	−2.05	−6.89	59.28
E3%-uB-15	18.50	0.41	0.09	−58.63	−1.91	−6.84	59.06
E3%-uB-30	18.30	0.28	−0.51	−58.84	−2.04	−7.44	59.34
E3%-uB-45	21.85	0.35	−0.65	−55.28	−1.97	−7.58	55.84
E3%-uB-60	20.85	0.37	−0.77	−56.29	−1.95	−7.70	56.85
E3%-uB-75	24.62	0.45	−0.97	−52.52	−1.87	−7.90	53.14
N0%-uA-ref	76.59	2.80	7.70				
N0%-uA-soot	22.00	0.57	1.34	−54.59	−2.24	−6.36	55.01
N0%-uA-15	22.23	0.59	1.32	−54.36	−2.22	−6.37	54.77
N0%-uA-30	22.82	0.62	1.33	−53.77	−2.19	−6.37	54.19
N0%-uA-45	20.09	0.45	1.14	−56.50	−2.35	−6.56	56.93
N0%-uA-60	21.46	0.60	1.47	−55.13	−2.21	−6.23	55.53
N0%-uA-75	22.01	0.59	1.28	−54.58	−2.22	−6.42	55.00
N0%-uB-ref	80.80	3.46	8.92				
N0%-uB-soot	21.24	0.77	1.52	−59.55	−2.69	−7.41	60.07
N0%-uB-15	20.45	0.76	1.33	−60.35	−2.70	−7.60	60.89
N0%-uB-30	21.64	0.90	1.52	−59.16	−2.56	−7.40	59.67
N0%-uB-45	22.55	1.05	1.90	−58.25	−2.41	−7.03	58.72
N0%-uB-60	19.49	0.67	1.23	−61.31	−2.79	−7.70	61.85

N0%-uB-75	22.63	0.95	1.69	−58.16	−2.52	−7.24	58.67
N0.5%-uA-ref	77.29	2.79	7.18				
N0.5%-uA-soot	21.74	0.61	0.87	−55.55	−2.18	−6.31	55.95
N0.5%-uA-15	23.25	0.77	0.61	−54.04	−2.02	−6.57	54.48
N0.5%-uA-30	24.12	0.85	0.56	−53.17	−1.94	−6.62	53.61
N0.5%-uA-45	24.02	0.67	0.25	−53.27	−2.12	−6.92	53.76
N0.5%-uA-60	27.64	0.91	0.54	−49.65	−1.88	−6.63	50.13
N0.5%-uA-75	34.20	0.80	0.20	−43.09	−1.99	−6.98	43.70
N0.5%-uB-ref	76.21	2.92	8.79				
N0.5%-uB-soot	19.35	0.27	0.60	−56.85	−2.64	−8.19	57.50
N0.5%-uB-15	25.77	0.63	−0.09	−50.44	−2.28	−8.87	51.26
N0.5%-uB-30	28.32	0.82	0.33	−47.88	−2.09	−8.46	48.67
N0.5%-uB-45	31.51	0.67	0.56	−44.69	−2.25	−8.23	45.50
N0.5%-uB-60	31.24	0.71	0.18	−44.97	−2.21	−8.61	45.84
N0.5%-uB-75	34.20	0.80	0.20	−42.00	−2.11	−8.59	42.92
N1%-uA-ref	78.60	3.21	7.51				
N1%-uA-soot	21.41	0.73	0.83	−57.19	−2.48	−6.68	57.63
N1%-uA-15	22.80	0.93	0.62	−55.80	−2.28	−6.90	56.27
N1%-uA-30	24.18	0.90	0.06	−54.42	−2.31	−7.45	54.97
N1%-uA-45	25.88	0.82	−0.15	−52.72	−2.39	−7.67	53.33
N1%-uA-60	29.40	1.44	−0.66	−49.20	−1.78	−8.17	49.91
N1%-uA-75	32.93	1.19	−0.91	−45.67	−2.03	−8.43	46.48
N1%-uB-ref	75.94	2.73	7.29				
N1%-uB-soot	19.32	0.28	0.30	−56.62	−2.45	−6.99	57.10
N1%-uB-15	20.53	0.30	−0.52	−55.41	−2.43	−7.81	56.01
N1%-uB-30	23.87	0.31	−0.96	−52.07	−2.42	−8.25	52.77
N1%-uB-45	28.54	0.30	−1.35	−47.40	−2.43	−8.64	48.24
N1%-uB-60	30.59	0.34	−1.35	−45.34	−2.39	−8.64	46.22
N1%-uB-75	35.03	0.57	−0.74	−40.91	−2.16	−8.03	41.74
N3%-uA-ref	75.69	3.69	9.02				
N3%-uA-soot	20.13	0.26	−1.08	−55.56	−3.43	−10.10	56.58
N3%-uA-15	22.29	0.42	−2.16	−53.40	−3.27	−11.18	54.66
N3%-uA-30	26.03	0.47	−2.83	−49.66	−3.22	−11.86	51.15
N3%-uA-45	31.31	0.44	−4.02	−44.38	−3.25	−13.05	46.37
N3%-uA-60	34.54	0.50	−3.27	−41.15	−3.20	−12.29	43.06
N3%-uA-75	40.13	0.77	−3.90	−35.56	−2.92	−12.92	37.94
N3%-uB-ref	75.55	3.14	8.10				
N3%-uB-soot	24.47	0.25	−1.09	−51.08	−2.90	−9.19	51.98
N3%-uB-15	37.45	0.50	−1.29	−38.11	−2.64	−9.39	39.33
N3%-uB-30	51.08	0.72	−2.83	−24.47	−2.42	−10.92	26.91
N3%-uB-45	57.25	1.20	−1.75	−18.30	−1.95	−9.85	20.87
N3%-uB-60	61.95	0.69	−2.16	−13.60	−2.45	−10.26	17.21
N3%-uB-75	63.42	1.21	−1.33	−12.13	−1.94	−9.43	15.49
